# Integrated physical, cognitive and psychological rehabilitation following multiple ACL reconstructions in a professional handball player: a case report

**DOI:** 10.1186/s13256-026-06732-2

**Published:** 2026-10-13

**Authors:** Marc Niering, Jana Goldstein, Rainer Beurskens, Elisa Ueding, Johanna Seifert

**Affiliations:** 1 Institute of Biomechanics and Neurosciences, Nordic Science, Hannover, Germany; 2 School of Sports, Psychology and Education, Triagon Academy Malta, Marsa, Malta; 3 Department of Health and Social Affairs, FHM Bielefeld-University of Applied Sciences, Bielefeld, Germany; 4 Institute of Sports Science, Faculty of Humanities, Leibniz University Hannover, Hannover, Germany; 5 Department of Psychiatry, Social Psychiatry and Psychotherapy, Hannover Medical School, Carl-Neuberg Straße 1, 30625 Hannover, Germany

**Keywords:** Anterior cruciate ligament injury, Psychological readiness, Neuromuscular function, Professional handball, Case report

## Abstract

**Background:**

Anterior cruciate ligament (ACL) injuries with repeated surgical interventions and postoperative complications pose a major challenge in sports rehabilitation. Beyond neuromuscular deficits, psychological and cognitive readiness often remain impaired, increasing reinjury risk and threatening athletic careers. This case highlights an integrated rehabilitation strategy addressing physical, psychological, and cognitive domains in a professional athlete with a complex surgical history.

**Case presentation:**

A 31-year-old white Serbian male professional handball player with a history of two ACL reconstructions, meniscal repairs, a postoperative knee infection, and multiple arthroscopic interventions presented with persistent knee instability, pain, and psychological distress. Following repeated failed return-to-play attempts, he underwent a ten-week intervention combining therapeutic exercises, sports psychology sessions, and neurocognitive dual-task training. Pre- and post-intervention assessments included gait analysis, postural control, strength testing, ACL-specific functional tests, and psychological evaluations. Post-intervention assessments showed improvements in gait mechanics, postural stability, neuromuscular strength, and reduced functional asymmetries, alongside lower cognitive anxiety and greater motivation.

**Conclusions:**

This case suggests that an interdisciplinary, multimodal rehabilitation approach, integrating physical, psychological, and cognitive strategies, may help address both physical deficits and psychological barriers in athletes with multiple ACL surgeries and complex postoperative courses. Such integration may support function, confidence, and readiness in high-performance athletes facing career-threatening injuries.

**Supplementary Information:**

The online version contains supplementary material available at https://doi.org/10.1186/s13256-026-06732-2.

## Introduction

Handball is classified as a high-intensity contact sport, characterized by frequent physical contact and demanding physical requirements, such as directional changes, speed adjustments, and divided attention [1–3]. These attributes increase the risk of injuries, particularly of ACL rupture, which is among the most common sports-related injuries in both amateur and professional athletes [4–8].

ACL injuries are often accompanied by joint effusion, pain, mechanical instability, and deafferentation due to the loss of mechanoreceptors, leading to central nervous system reorganization and manifesting as deficits in proprioception and motor coordination [9]. These sensorimotor impairments result in altered proprioceptive feedback, decreased neuromuscular control, and increased reliance on cognitive and visual processing during motor tasks, thereby necessitating targeted strength, neuromuscular and cognitive interventions [10, 11]. ACL reconstruction is a standard orthopedic procedure aimed at restoring knee stability and preventing associated injuries [12, 13], with the primary goal being a return to competition (RTC) at pre-injury performance levels [14]. However, the factors influencing successful RTC are not fully understood. Although ample research exists on treating strength deficits, neuromuscular control, and clinical function post-ACL reconstruction, the psychological components impacting RTC remain underexplored [4].

For professional athletes, an ACL injury can be career-altering, accompanied by significant psychological trauma and an increased likelihood of failing to return to elite competition [15]. Studies indicate that beyond neuromuscular function, psychological readiness—including cognitive, emotional, and behavioral responses—is crucial for RTC [16, 17]. Psychosocial factors such as hesitation, fear, and lack of confidence can hinder the recovery process [18].

Contact sports athletes are particularly vulnerable to reinjury due to neuromuscular and neurocognitive deficits following ACL rupture [9]. The decision to return to sport is multifactorial, influenced by both physical metrics and environmental and psychosocial factors. Given the gap between functional knee outcomes and RTC rates, addressing psychological factors is essential [4]. The present case of a professional handball player, who underwent two ACL reconstructions with multiple complications, represents a rare scenario with limited data [4, 17]. This study’s unique approach combines therapeutic training and sports psychology interventions to optimize rehabilitation and support a return to high-level sport. The intervention integrates dual-task exercises [3], attention direction, emotion regulation [1], and mindset development, alongside correction of neuromuscular imbalances [17].

This case is clinically relevant because it demonstrates how the integration of psychological interventions into a multimodal rehabilitation program can enhance neuromuscular function, psychological readiness, and ultimately the likelihood of returning to high-level competition.

## Methods

This case report was prepared and reported in accordance with the CARE (CAse REport) guidelines [19] (Supplement 2).

The baseline assessment was conducted on June 27, 2022, before the start of the intervention. The post-intervention assessment was conducted on September 8, 2022, seven days after the final supervised session on September 1, 2022. All pre- and post-intervention physical assessments were performed by the same examiner (MN).

### Case presentation

**Fig. 1:**
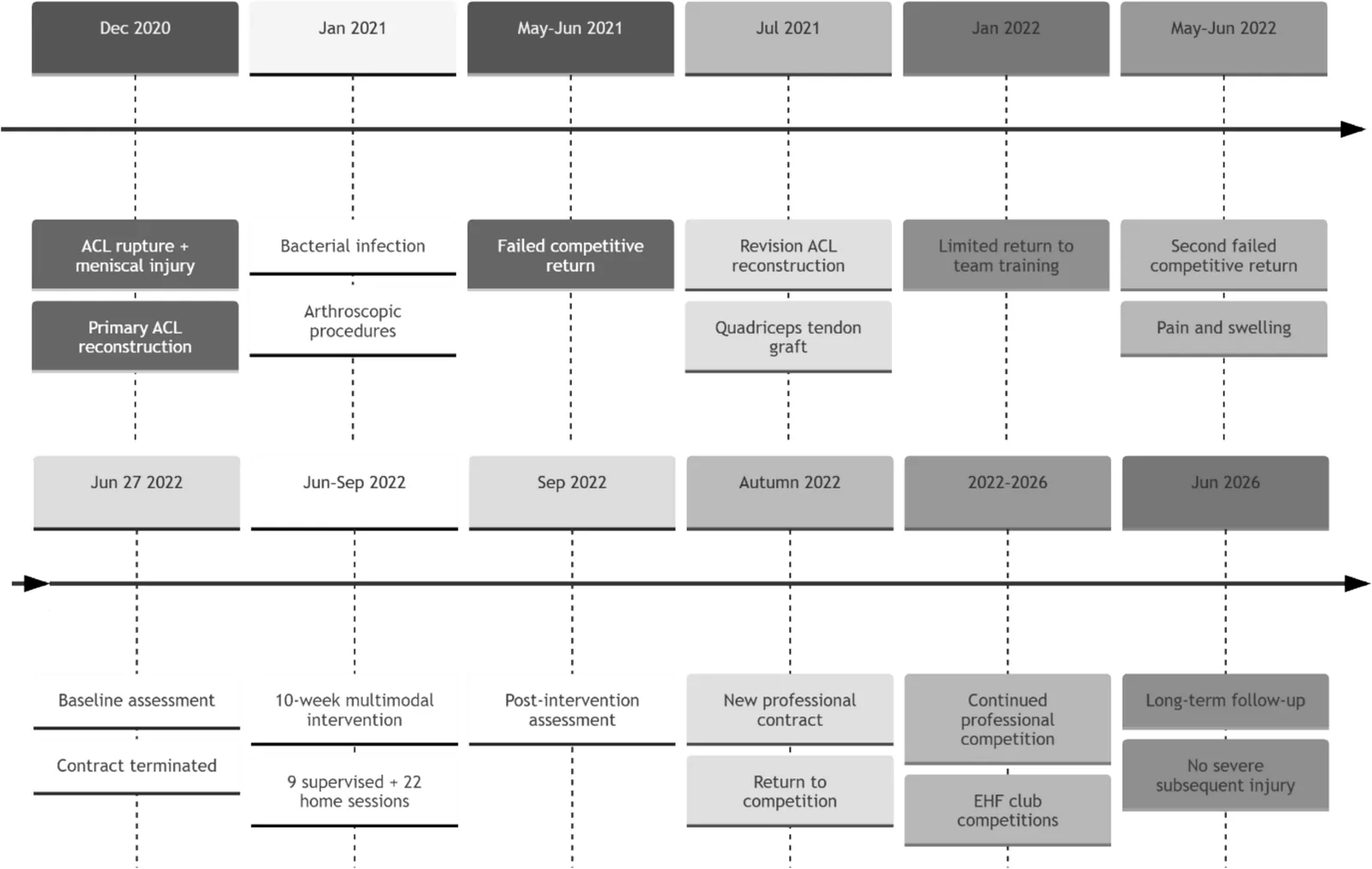
Medical history timeline

The athlete, a 31-year-old white Serbian male professional handball player, serves as captain and plays defense in the left backcourt. His medical history (Fig. 1) began in December 2020 with an ACL rupture of the right knee, accompanied by a lateral meniscus lesion and a ramp lesion in the medial meniscus. Surgical intervention included meniscus refixation with smoothing and ACL reconstruction using a four-strand semitendinosus graft. In January 2021, a bacterial infection in the right knee was identified, leading to the arthroscopic insertion of a Sulmycin sponge. On January 30, 2021, medication carriers were inserted, and the implant was removed arthroscopically.

Between May and June 2021, the player attempted to participate in three games but had to leave each after a maximum of ten minutes due to feelings of instability and subsequent swelling. In July 2021, the ACL was examined under anesthesia, and reconstruction was performed using a quadriceps tendon graft. Postoperative care and early rehabilitation were supported by standard physical therapies following each procedure to facilitate his return to sport.

At the time of data collection, the athlete reported a sensation of instability in the right knee, along with morning pain localized around the tibial plateau at the level of the screws. He described feeling weak, fatigued, and experiencing numbness around the right knee. Movement reportedly provided some relief, though it led to a visible compensatory load on the left side, confirmed by video analysis. Observationally, the athlete displayed an upright posture with a slight right scapular elevation.

In January 2022, he re-entered training on a limited basis. Following an extended contract through the summer, the goal was to contribute to the team’s league retention. However, during three games between May and June, he managed only two sessions of ten minutes each due to recurring knee pain and swelling in the right knee afterward.

By the initial diagnostic evaluation, his contract with the club had already been terminated, necessitating the return of club-provided sports equipment, his vehicle, and his apartment, adding psychological strain. He expressed uncertainty about his career and reported frequently ruminating on his knee condition. He was considering ending his athletic career to pursue academic studies, basing his final decision on the test results and external evaluations from the therapists and researchers conducting the intervention. Furthermore, the athlete actively sought the support of a sports psychologist, expressing concerns about his mental health and recognizing its critical role in his rehabilitation process.

### Physical testing procedures

The test battery included assessments of passive joint mobility, gait and running analysis, postural control, dynamic and isometric muscle strength, and ACL-specific tests [17]. Selected tests were performed using VALD ForceDecks (VALD Performance, Brisbane, Australia) for side-to-side comparison. All assessments were conducted bilaterally and barefoot to ensure natural foot contact. Symmetry was evaluated using the Limb Symmetry Index (LSI), with values <90% indicating relevant functional deficits [20]. Test-specific reference values from the modular return-to-play test battery described by Bloch et al. [21] were used to interpret the assessment results. These included an LSI of at least 85% for isokinetic maximum strength and a Single Leg Hop for Distance of 80–90% of body height with a side difference of no more than 20 cm. As some assessments used in the present case differed from the corresponding procedures described by Bloch et al., including isometric rather than isokinetic strength testing, these values were used as reference values for interpreting functional changes and not as stand-alone return-to-competition clearance criteria. The assessments were conducted in the following order: passive knee joint examination, video-based gait and running analysis, postural-control testing, squat and jump testing, isometric strength testing, and ACL-specific hop testing. For all strength, jump, and hop assessments, four recorded trials were completed with 30-s rest intervals, and the best trial was used for analysis.

Passive knee joint mobility was assessed using goniometry, including flexion, extension, and end-feel evaluation [7, 13]. These measures helped identify movement restrictions and asymmetries relevant to rehabilitation.

Gait and running mechanics were assessed using video-based qualitative analysis recorded with a Sony ZV-1 camera (Sony Corporation, Tokyo, Japan) to identify asymmetries, loading patterns, and compensatory movements relevant to ACL rehabilitation [22]. The athlete was observed on a treadmill and short track under walking and running conditions. Attention focused on step length, cadence, limb symmetry, and trunk posture.

Postural control and proprioception were assessed using standardized balance tests to detect asymmetries and instability relevant to ACL rehabilitation [9, 23]. The Y-Balance Test (YBT) was applied to evaluate dynamic balance and functional asymmetry in the lower limbs [24]. Performance was recorded as composite scores relative to leg length, with lower scores indicating impaired balance. YBT results were interpreted descriptively and were not used as a stand-alone RTC clearance criterion. Additional assessments on force plates included quiet standing balance (bilateral/unilateral, eyes open and closed) and a motor dual-task variant with ball catching to simulate sport-specific demands [25]. A single-leg stance test was also performed to evaluate postural stability.

Dynamic strength was evaluated using squats, single-leg squats, countermovement jumps (CMJ), and drop jumps (DJ) [26].

These tests assessed lower limb strength, balance, postural control, and explosive power relevant to ACL rehabilitation [17, 27]. Performance was recorded bilaterally with attention to symmetry and movement quality.

Isometric muscle strength was assessed using the VALD ForceFrame system (VALD Performance, Brisbane, Australia), targeting knee flexion/extension and hip flexion/extension at 90° joint angles [28]. These measures provided information on leg strength, a critical factor for return to competition and movement quality [29].

To assess the injury in greater detail, several hop tests were conducted. The Side Hop Test evaluated functional strength under fatigue and lateral asymmetries [30]. The Single Leg Hop for Distance together with its triple and multiple variants measured functional hop performance and side-to-side asymmetry [27, 31, 32].

The Cross Over Hop Test assessed jump performance and side-to-side asymmetry [26, 33].

### Psychological testing procedures

In addition to neuromuscular impairments, ACL rupture affects psychological readiness [17]. Therefore, psychological assessments were conducted using the Competitive Anxiety Inventory (WAI-T) [34], the Perceived Physical State Questionnaire (WKV-20) [35], and the Eigenzustandsskala (EZK) [36]. The WAI-T evaluated competition-related anxiety, the WKV-20 assessed perceived physical condition, and the EZK captured situational well-being. These assessments helped to identify deficits requiring targeted interventions [37]. The same psychological assessments were administered by MN at baseline and at the post-intervention assessment on September 8, 2022. No standardized psychological assessments had been conducted during the athlete’s previous surgical or rehabilitation history before the baseline assessment of the present case. The semi-structured interview used to document the patient perspective was conducted by MN on September 8, 2022, following the post-intervention assessment.

### Combined physical, cognitive and psychological intervention

The intervention consisted of nine supervised sessions over ten weeks, each lasting 60 min, complemented by 22 home sessions documented in a training diary [38]. All supervised sessions were delivered by a sports physiotherapist who was not involved in data analysis or manuscript preparation. The number of supervised sessions reflected the treatment funding available for the case and the ten-week period available before the athlete’s planned professional tryouts. Because his previous club contract had expired, participation in team training was not possible during the intervention for insurance-related reasons. Handball-specific exposure was therefore limited to one-on-one practice with his brother, and professional tryouts took place only after completion of the intervention and the final RTC assessment. The home sessions comprised the same strength, postural-control, and plyometric exercises used during the supervised sessions. External load progression was based on the athlete’s perceived difficulty and movement quality rather than on a predefined percentage increment. Adherence to the home program was monitored using the training diary. The specific exercises, training volume, load prescription, movement tempo, and recovery periods are presented in Supplementary Figure S1.

A sports psychologist (MN) conducted an initial 30-min session focusing on mindset, body image, and sports-specific risks to strengthen self-efficacy and psychological readiness [5, 39]. Before the intervention, the sports physiotherapist received three 15-min coaching sessions from the sports psychologist covering positive self-instruction, goal setting, cognitive restructuring, graded exposure, and motor imagery. Throughout the subsequent training, the sports physiotherapist reinforced these contents by applying cognitive-behavioral strategies such as medical education, positive affirmations, goal setting, cognitive restructuring, graded exposure, and motor imagery. This approach ensured that psychological and physical rehabilitation were addressed in parallel and is consistent with evidence showing that psychological interventions can be delivered by appropriately trained physiotherapists [40]. The physical and psychological contents were individualized according to the deficits identified during the baseline assessment and the movements and situations that the athlete had described as provoking insecurity or anxiety.

Each session began with a short treadmill warm-up and dynamic exercises, followed by dual-task drills (e.g., ball catches, kettlebell swings) and agility tasks with visual cues to add neurocognitive load. This approach was based on recent evidence showing that unanticipated or cognitively demanding tasks reveal persistent coordination deficits after ACL reconstruction that standard tests may miss, thereby justifying their systematic integration into rehabilitation [41, 42]. Stance training with varied surfaces and head-eye gaze shifts [9] targeted proprioceptive control, while functional strength exercises addressed neuromuscular imbalances [8].

Plyometric drills, including the Single Leg Drop Jump, and agility tasks with visual cues enhanced stretch–shortening capacity and sport-specific coordination [32, 43].

During the final three sessions, high-intensity interval sprints with added cognitive load simulated competitive fatigue conditions, aiming to build resilience and situational awareness [44]. Each block consisted of four 20-s maximal sprints on a non-motorized treadmill (Sprintbok, NOHrD, Germany), targeting 90–95% of maximum heart rate. Heart rate was recorded continuously using a Polar H10 chest-strap monitor (Polar Electro Oy, Kempele, Finland). Maximum heart rate was defined as the highest heart rate recorded during a maximal-effort test performed before the intervention. Immediately after each sprint, the athlete stepped off the treadmill and performed color-based reactive stepping exercises combined with cognitive dual-tasks and movement patterns that he had previously described as anxiety-provoking. These included a handball-specific blocking movement involving a plant step with the affected leg. The reactive exercises continued until heart rate decreased below 70% of maximum heart rate, after which the next sprint began. After four sprint-reaction cycles, the athlete rested for 60 s. This block was completed three times, resulting in twelve 20-s sprints per session. Session rating of perceived exertion was assessed at the end of each session using the Borg 6–20 scale, with a target session RPE of 18–20 [45]. The target range was reached in each of the three sessions, although the exact session-specific RPE values were not retained.

### Return-to-competition outcome and long-term follow-up

For the present case, successful return to competition was defined as participation in a professional competitive handball match after completion of the intervention. Contract signing, participation in individual training, or return to team training alone was not considered sufficient to establish successful return to competition. Long-term follow-up information on continued professional competition, current club affiliation, and subsequent injuries that had interrupted or ended professional participation was obtained from the athlete in June 2026.

## Results

Post-intervention assessments showed improvements in both physical and psychological domains. Physical outcomes included enhanced gait mechanics, postural control, functional performance, muscle strength, and hop capacity. Psychological outcomes reflected reduced cognitive anxiety, improved motivation, and perceived stress tolerance. Detailed numerical results are provided in Supplementary Tables S1–S6 (Supplement 1).

### Physical measures

The athlete’s gait improved from a short-stride, toe-running pattern with reduced right-arm swing to a dynamic midfoot strike with reactive arm movement. The initial divergence and circumduction of the right leg, with external tibial rotation and internal hip rotation, were no longer observed at post-intervention assessment. The Trendelenburg sign during stance was no longer observed at post-intervention assessment, and posterior pelvic tilt was reduced. At post-intervention assessment, static balance on the injured leg showed greater stability and fewer compensatory trunk movements than at baseline. Dynamic balance under perturbations and dual-task conditions also showed greater stability at post-intervention assessment than at baseline. Functional performance also differed between baseline and post-intervention assessment: the deep squat changed from limited depth and asymmetrical weight-bearing to symmetrical execution with full depth, while the single-leg squat improved from valgus collapse and trunk instability to stable movement control. Vertical jump outcomes showed greater symmetry at post-intervention assessment, and the reactive strength index increased. Knee-flexion and knee-extension LSIs increased from 51.5% to 90.5% and from 67.8% to 87.4%, respectively, thereby exceeding the numerical strength reference value of ≥85%. Hop-test LSIs increased, although not all individual limb values improved. Not all test-specific reference values were met, as the Single Leg Hop for Distance showed a residual side difference of 23 cm. During the bilateral drop jump, the eccentric mean-force LSI remained at 70.0%. At the final RTC assessment, symptoms were minimal and did not prevent completion of the test battery. Complete results are presented in Supplementary Tables S1-S5.

### Psychological measures and patient’s perspective

Psychological testing showed reductions in cognitive anxiety from 14 to 12 points and concentration disruptions from 9 to 8 points, while somatic anxiety remained unchanged at 10 points. Other measures showed mixed changes, including increases in alertness, overall motivation, flexibility, and stress tolerance, decreases in readiness to engage and mood, and unchanged pain (detailed results in Table S6, Supplement 1).

At the post-intervention interview, the athlete reported greater perceived security, confidence, and readiness to return to professional sport. He described the program as challenging yet purposeful, emphasizing that the psychological strategies learned (e.g., positive self-instruction, goal setting, cognitive restructuring) empowered him to face demanding situations with greater resilience. Despite the uncertainty of being without a club, he no longer experienced fear of the future and approached upcoming tryouts with optimism. Following the final RTC assessment, the rehabilitation team provided clinical clearance for a return to professional handball. The final decision to return was made by the athlete himself. During a consultation with the insurer, he declined an offer of a funded sports-management degree and decided to pursue a return to professional competition despite the absence of a confirmed contract. Three months later, he signed a new contract and successfully competed in the EHF European League. At the long-term follow-up in June 2026, the athlete reported that he continued to compete for the same club without a subsequent injury that had interrupted or ended his professional participation.

## Discussion

The physical assessments showed changes in symmetry and balance across postural, functional, isometric, and ACL-specific tests. Postural control and strength outcomes generally improved, consistent with evidence that task-specific training can enhance stability and symmetry [22, 46, 47].

High-intensity eccentric and plyometric drills may have contributed to the observed functional gains, consistent with Kasmi et al. [32]. Young et al. [43] further demonstrated that dual-task training can foster an adaptive stretch–shortening cycle (SSC), vital for competitive performance.

The psychological effects were notable, while changes under dual-task conditions were limited to postural-control outcomes [9]. Sport-specific stressors simulated game demands [37]. This aligns with the EZK results and may indicate changes in perceived stress tolerance. However, the clinical relevance of these changes remains uncertain, and they may also have been influenced by concurrent physical improvements and contextual factors related to the athlete’s professional future and upcoming tryouts. Though somatic anxiety remained unchanged, reductions in cognitive anxiety and concentration disturbances in the WAI-T test may reflect fewer cognitive and attentional barriers.

This case supports Piussi et al. [18], who noted increased nervousness after re-injury, highlighting the relevance of psychological factors to perceived readiness to RTC. Consistent with previous findings [48], integrating psychological support with physical rehabilitation may support psychological readiness, although effects on reinjury risk cannot be determined from the present case.

These findings are consistent with ACL-specific literature emphasizing that rehabilitation should integrate attentional focus, motor-learning, and dual-task principles to directly address the neurophysiological and neuroplastic alterations following ACL rupture. Such approaches are reflected in our integrated protocol, which simultaneously targeted psychological, cognitive, and physical domains rather than treating them as separate components [49, 50].

The integrated principles used in the present case may also be relevant to other multidirectional team sports that require rapid decision-making under fatigue. They may further be applicable to athletes with prolonged or recurrent lower-limb injuries accompanied by insecurity, fear of reinjury, or reduced confidence. However, their transferability to other sports, injuries, and athlete populations requires further investigation.

This case study has several limitations. As a single case, comparison with similar patients was not feasible, and the multimodal intervention makes it difficult to isolate the effects of individual components [18]. The mindset component was delivered by the sports psychologist in a single session. Although its content was reinforced by the physiotherapist during subsequent supervised sessions, no further sessions were conducted by the sports psychologist, which may have limited the intensity of this component. The ten-week intervention and the limited number of supervised sessions restricted the assessment of dose–response relationships and the persistence of effects attributable to individual intervention components. As baseline assessment was conducted approximately eleven months after the revision ACL reconstruction and no control condition was available, natural recovery and the effects of previous or ongoing rehabilitation cannot be excluded. Collaboration with the club was limited after contract termination, which reduced opportunities for full sport-specific reintegration.

## Conclusion

This case study suggests that combining therapeutic exercise with sports psychology may be beneficial in a professional athlete with repeated ACL injuries. The integrated approach was accompanied by improvements in gait mechanics, postural control, muscle strength, functional performance, and psychological factors such as motivation and competition anxiety.

Although not all parameters changed, improvements in psychological readiness and resilience were observed alongside neuromuscular recovery. This underlines the importance of addressing cognitive, emotional, and physical factors simultaneously in rehabilitation.

For clinical practice this case provides a key message that systematically integrating psychological strategies into physiotherapy may support both function and confidence and thereby support safe return to elite competition. Larger studies are warranted to further examine the interaction of psychological and neuromuscular factors in high-performance athletes.

## Supplementary Information

Additional file 1

Additional file 2

## Data Availability

The original contributions presented in the study are included in the article/supplementary material. Further inquiries can be directed to the corresponding author/s.

## Abbreviations

ACL: Anterior cruciate ligament
RTC: Return to competition
CARE: CAse REport guidelines
LSI: Limb Symmetry Index
YBT: Y-Balance test
DJ: Drop jump
WAI-T: Competitive anxiety inventory
WKV-20: Perceived Physical State Questionnaire
EZK: Eigenzustandsskala
SSC: Stretch–shortening cycle
