# Supplementary material for: Integrated physical, cognitive and psychological rehabilitation following multiple ACL reconstructions in a professional handball player: a case report
Source: J Med Case Rep. 2026 Oct 13;20:621. doi: 10.1186/s13256-026-06732-2 (PMC13663265; doi:10.1186/s13256-026-06732-2)
Supplement: Supplementary file 1 [file 13256_2026_6732_MOESM2_ESM.pdf]

CARE case report guidelines

CARE Checklist of information to include when writing a case report

(cc) BY-NC-ND

Topic	Item	Checklist item description	Reported on Line
Title	1	The diagnosis or intervention of primary focus followed by the words "case report"	1-2
Key Words	2	2 to 5 key words that identify diagnoses or interventions in this case report, including "case report"	69-70
Abstract (no references)	3a	Introduction: What is unique about this case and what does it add to the scientific literature?	47-52
	3b	Main symptoms and/or important clinical findings	53-62
	3c	The main diagnoses, therapeutic interventions, and outcomes	53-62
	3d	Conclusion—What is the main "take-away" lesson(s) from this case?	63-67
Introduction	4	One or two paragraphs summarizing why this case is unique (**may include references**)	118-131
Patient Information	5a	De-identified patient specific information.	140-141
	5b	Primary concerns and symptoms of the patient.	157-174
	5c	Medical, family, and psycho-social history including relevant genetic information	140-174
	5d	Relevant past interventions with outcomes	143-166
Clinical Findings	6	Describe significant physical examination (PE) and important clinical findings.	157-162, 308-327
Timeline	7	Historical and current information from this episode of care organized as a timeline	144-156 (Fig. 1)
Diagnostic Assessment	8a	Diagnostic testing (such as PE, laboratory testing, imaging, surveys).	175-237
	8b	Diagnostic challenges (such as access to testing, financial, or cultural)	NA
	8c	Diagnosis (including other diagnoses considered)	140-151
	8d	Prognosis (such as staging in oncology) where applicable	NA
Therapeutic Intervention	9a	Types of therapeutic intervention (such as pharmacologic, surgical, preventive, self-care)	238-293
	9b	Administration of therapeutic intervention (such as dosage, strength, duration)	239-293
	9c	Changes in therapeutic intervention (with rationale)	248-253, 277-293
Follow-up and Outcomes	10a	Clinician and patient-assessed outcomes (if available)	301-348
	10b	Important follow-up diagnostic and other test results	301-334
	10c	Intervention adherence and tolerability (How was this assessed?)	239-253, 290-293
	10d	Adverse and unanticipated events	NA
Discussion	11a	A scientific discussion of the strengths AND limitations associated with this case report	349-394
	11b	Discussion of the relevant medical literature **with references**.	349-381
	11c	The scientific rationale for any conclusions (including assessment of possible causes)	349-381
	11d	The primary "take-away" lessons of this case report (without references) in a one paragraph conclusion	395-408
Patient Perspective	12	The patient should share their perspective in one to two paragraphs on the treatment(s) they received	329-340
Informed Consent	13	Did the patient give informed consent? Please provide if requested	Yes ☒ No ☐
