# Supplementary material for: Integrated physical, cognitive and psychological rehabilitation following multiple ACL reconstructions in a professional handball player: a case report
Source: J Med Case Rep. 2026 Oct 13;20:621. doi: 10.1186/s13256-026-06732-2 (PMC13663265; doi:10.1186/s13256-026-06732-2)
Supplement: Supplementary file 2 [file 13256_2026_6732_MOESM1_ESM.docx]

**Supplementary Material**

Integrated physical, cognitive and psychological rehabilitation following multiple ACL reconstructions in a professional handball player: a case report

**Table S1.** Postural control results.

**Table S2.** Squat results.

**Table S3.** Drop Jump results.

**Table S4.** Isometric strength results.

**Table S5.** ACL-specific results.

**Table S6.** Psychological results.

**Figure S1.** Home exercises.

**Table S1.** Postural control results

| A. Y-Balance Test | | Composite score (%) | | | LSI (%) |  |
| --- | --- | --- | --- | --- | --- | --- |
|  |  | left | | Right |  |  |
| Y-Balance Test | Pre | 94.6 | | 88.3 | 93.3 |  |
|  | Post | 101.5 | | 91.3 | 90.0 |  |
|  | **Δ** | 6.9 | | 3.0 | **-3.3** |  |
| B. Force-plate balance assessment | | Total excursion [mm] | | | Total excursion LSI (%) | Mean force  LSI (%) |
|  |  | left | right | |  |  |
| Quiet standing balance | Pre | 137 | 346 | | 39.6 | 93.8 |
|  | Post | 101 | 126 | | 80.2 | 97.4 |
|  | **Δ** | -36 | -220 | | **+40.6** | **+3.6** |
| Quiet standing balance eyes closed | Pre | 151 | 220 | | 68.6 | 95.8 |
|  | Post | 141 | 165 | | 85.5 | 96.1 |
|  | **Δ** | -10 | -55 | | **+16.8** | **+0.3** |
| Quiet standing balance dual-task | Pre | 515 | 850 | | 60.6 | 84.7 |
|  | Post | 327 | 390 | | 83.8 | 96.5 |
|  | **Δ** | -188 | -460 | | **+23.3** | **+11.8** |
| Single leg standing balance | Pre | 626 | 901 | | 69.4 |  |
|  | Post | 552 | 625 | | 88.3 |  |
|  | **Δ** | -74 | -276 | | **+18.9** |  |

*Note:* LSI = limb symmetry index.

**Table S2.** Squat results

| Test | Peak Force in [N] | | | | | | Peak Force LSI (%) | | |
| --- | --- | --- | --- | --- | --- | --- | --- | --- | --- |
|  | left | | | right | | |  |  |  |
|  | Pre | Post | Δ | Pre | Post | Δ | Pre | Post | **Δ** |
| Squat | 832.5 | 872.2 | +39.7 | 623.1 | 740.8 | +117.7 | 74.8 | 84.9 | **+10.1** |
| Single leg squat | 1380.1 | 1440.5 | +60.4 | 1090.2 | 1381.4 | +291.2 | 79.0 | 95.9 | **+16.9** |
| CMJ | 1498.2 | 1568.9 | +70.7 | 1210.0 | 1551.7 | +341.7 | 80.8 | 98.9 | **+18.1** |

*Note:* CMJ = counter movement jump; LSI = limb symmetry index.

**Table S3**. Drop Jump results

| Test |  | RSI | Concentric mean force LSI (%) | Eccentric mean force LSI (%) | Landing RFD LSI (%) |
| --- | --- | --- | --- | --- | --- |
| Drop Jump | Pre | 0.88 | 76.0 | 63.0 | 86.0 |
|  | Post | 1.04 | 96.8 | 70.0 | 94.0 |
|  | **Δ** | **+0.16** | **+20.8** | **+7.0** | **+8.0** |

Note: RSI = reactive strength index; LSI = limb symmetry index; RFD = rate of force development

**Table S4.** Isometric strength results

| Test | Peak Force [N] | | | | | | Peak Force LSI (%) | | |
| --- | --- | --- | --- | --- | --- | --- | --- | --- | --- |
|  | left | | | right | | |  |  |  |
|  | Pre | Post | Δ | Pre | Post | Δ | Pre | Post | **Δ** |
| Knee flexion | 276.8 | 310.2 | +33.5 | 142.5 | 280.6 | +138.1 | 51.5 | 90.5 | **+39.0** |
| Knee extension | 384.5 | 391.3 | +6.8 | 260.5 | 342.0 | +81.6 | 67.8 | 87.4 | **+19.7** |
| Hip flexion | 404.6 | 426.2 | +21.6 | 375.5 | 395.7 | +20.2 | 92.8 | 92.8 | **0** |
| Hip extension | 268.8 | 391.6 | +122.8 | 217.0 | 352.1 | +135.1 | 80.7 | 89.9 | **+9.2** |

*Note:* LSI = limb symmetry index.

**Table S5***.* ACL-specific results

| Test | | Distance [cm] | | Hops [n] | | LSI (%) |
| --- | --- | --- | --- | --- | --- | --- |
|  |  | left | right | left | right |  |
| Side hop (40cm) | Pre |  |  | 56 | 43 | 76.8 |
|  | Post |  |  | 48 | 43 | 89.6 |
|  | Δ |  |  | -8 | 0 | **+12.8** |
| Single leg hop for distance | Pre | 182 | 137 |  | | 75.3 |
|  | Post | 178 | 155 |  | | 87.1 |
|  | Δ | -4 | +18 |  | | **+11.8** |
| Triple single leg hop | Pre | 520 | 458 |  | | 88.1 |
|  | Post | 560 | 522 |  | | 93.2 |
|  | Δ | +40 | +64 |  | | **+5.1** |
| Cross- over hop | Pre | 470 | 400 |  | | 85.1 |
|  | Post | 500 | 472 |  | | 94.4 |
|  | Δ | +30 | +72 |  | | **+9.3** |

**Table S6.** Psychological results

| Domain/Test | WAI-T | | | | |
| --- | --- | --- | --- | --- | --- |
|  | Pre | | Post | | Δ |
| Somatic anxiety | 10 | | 10 | | 0.0% |
| Cognitive anxiety | 14 | | 12 | | -14.3% |
| Concentration disruptions | 9 | | 8 | | -11.1% |
|  | **WKV-20** | | | | |
| Alertness | 2.4 | | 3.0 | | +25.0% |
| Fitness | 3.8 | | 4.4 | | +15.8% |
| Flexibility | 2.6 | | 3.2 | | +23.1% |
| Health | 3.4 | | 4.4 | | +29.4% |
| Physical complaints | 4.0 | | 3.0 | | -25.0% |
| Pain level | 2.0 | | 2.0 | | 0.0% |
|  | **EZK** | | | | |
| Overall Motivation | 4.0 | 4.25 | | +6.3% | |
| Social approval | 3.0 | 4.0 | | +33.3% | |
| Self-confidence | 4.5 | 5.0 | | +11.1% | |
| Readiness to engange | 5.0 | 4.0 | | -20.0% | |
| Willingness to exert effort | 3.5 | 4.0 | | +14.3% | |
| Overall stress tolerance | 3.6 | 3.9 | | +8.3% | |
| Inner calm | 3.5 | 4.0 | | +14.3% | |
| Mood | 4.5 | 4.0 | | -11.1% | |
| Recovery | 3.5 | 4.5 | | +28.6% | |
| Restfulness | 3.0 | 3.0 | | 0.0% | |

**Figure S1.** Home Exercises.

*Note:* The physical exercises shown in the figure also formed the home-training program. External load progression was based on the athlete’s perceived difficulty and movement quality rather than on a predefined percentage increment.
